# Neuroscience and Consumer Behavior: Where to Now?

**DOI:** 10.3389/fpsyg.2021.705850

**Published:** 2021-07-05

**Authors:** Michela Balconi, Martina Sansone

**Affiliations:** ^1^International Research Center for Cognitive Applied Neuroscience, Catholic University of the Sacred Heart, Milan, Italy; ^2^Research Unit in Affective and Social Neuroscience, Department of Psychology, Catholic University of the Sacred Heart, Milan, Italy

**Keywords:** consumer neuroscience, brain, neuroeconomics, autonomic measures, EEG, fNIRS, neuroscientific tools

## The State of the Art and Some Unresolved Issues

Over the past 20 years, the interest in consumer neuroscience (CN) has grown exponentially. In a period of economic stagnation, it is little wonder that the possibility to uncover and capitalize on the understanding of the neural mechanisms responsible for purchase behavior has attracted the interest of many corporations (Blakeslee, 2004; Plassmann et al., 2012). When the combination of economic theories and neuroscience gave rise to the transdisciplinary field of Neuroeconomics, soon after, a sub-area of interest emerged related to economic behaviors. CN, indeed, aims to take advantage of the study of neural correlates underlying decision-making to understand which factors guide the consumer’s journey up to making a purchase decision.

However, the appeal of applying neuroscience to the field of consumer research has resulted sometimes in pressures to commercialize neuroscientific methods, as well as in over-statements and inappropriate claims on behalf of companies, which did not receive any support from scientific evidence (Lindstrom, 2008; Stanton et al., 2017). For this reason, and for methodological issues that will be discussed below, CN has long struggled to ascertain its credibility, facing alternate periods of euphoria and distrust toward neuroscience-based findings (Ariely and Berns, 2010). Hence, a few decades after its onset, CN is still considered to be a promising discipline in need of a stronger theoretical and methodological implant.

Neurophysiological tools, indeed, proved able to benefit classical consumer research because collecting measures of autonomic and central neural activation opened up the possibility to look into implicit processes that affect purchase behaviors, which cannot be identified by traditional market research methods, such as self-reports, interviews, or focus-groups (Groeppel-Klein, 2005). Since people generally lack awareness of emotional, attentional, and sensorial integration processes and are not able to retrospectively retrace them, the verbalization of the opinion of consumers usually leaves out implicit attitudes and, overall, fails to reflect the most authentic product appraisal (Bagozzi, 1991; Ohme et al., 2011; Walla et al., 2014; Li et al., 2018). Conversely, neuroscientific measures bear the advantage of allowing a real-time objective recording of neurophysiological and emotional responses, cleared of cognitive biases and socially desirable responses (Camerer et al., 2005; Groeppel-Klein, 2005; Balconi et al., 2014; Balconi, 2014; Leanza and Balconi, 2017).

On this trail, the first years of research activity in the field were mostly dedicated to exploratory studies that strived to uncover the neural underpinnings of the psychological processes that intervene in the consumer experience. These studies have been linked to the “*context of discovery*” (Hubert, 2010). The neural correlates of product preference (Erk et al., 2002; Paulus and Frank, 2003; Levy et al., 2011), brand familiarity and recall (McClure et al., 2004; Deppe et al., 2005), price evaluation (Knutson et al., 2007; Plassmann et al., 2007), and integration of affective and motivational components for products and advertisements (Plassmann et al., 2008; Vecchiato et al., 2011) were investigated, in an effort to identify how the activity of specific brain areas can predict purchase decision. In those years, neuroscience brought noticeable insights into the understanding of consumer behavior (Kenning et al., 2002; Deppe et al., 2005; Balconi et al., 2014).

However, the investigation on the role of localized brain regions, motivated by the assumption of a one-to-one relationship between one type of psychological process and a specific area in the brain, allegedly specialized in only that mental process, also led to some over-simplifications (Solnais et al., 2013). The inductive logic that characterized these studies has been considered to some extent a valuable tool within little-explored research contexts, as it promotes scientific discovery; however, several authors have warned against the systematic use of inductive logic because the reasoning proceeds in the opposite direction of what neuroscience generally does, resulting in the logical fallacy of “*reverse inference*” (Poldrack, 2006; Hubert, 2010).

The risk of reverse inference, together with the scarce validity enabled by exploratory, poorly rigorous, and small-scale studies, prompted authors to advocate a methodological turning-point that would allow CN to turn from the “*context of discovery*” to the “*theory testing*” phase (Hubert, 2010). In order to make its contribution valuable and relevant to the purpose of clarifying the dynamics of purchase behaviors, CN should, on the one hand, deploy its tools to test—and eventually disconfirm—theories from economic psychology, decision-making, and consumer science, that are still short of empirical validation. On the other hand, it should make greater use of rigorous methodology and compelling experimental designs and strive to reproduce and validate the results of previous studies on a larger scale. Indeed, the crisis of reproducibility (Poldrack et al., 2019) further questioned the already wavering credibility of the discipline.

In more recent years, technological advances in neuroscience, supported by the extensive development of software programs and computer processing, as well as by new more portable and usable neuroscientific devices, allowed for honing of the neuroscientific techniques and maturation of the CN discipline. Neuroimaging techniques, which allow visualization of cerebral activity as a result of metabolic changes within the brain, attracted considerable interest in the field. Functional Magnetic Resonance Imaging (fMRI) has been extensively used because it allows localizing with excellent spatial resolution the activity of brain regions recruited during the experimental tasks (Levy and Glimcher, 2012). The primacy of fMRI has recently been complemented by functional Near Infra-Red Spectroscopy (fNIRS), a promising emerging technique which, like fMRI, but with more user-friendly applicability, can detect metabolic changes and can be used to map the cortical regions associated with ongoing psychological processes (Kopton and Kenning, 2014; Balconi and Molteni, 2016; Krampe et al., 2018). On the other hand, neuroscientific tools that measure cortical electrical activity, such as Electroencephalography (EEG), are excellent instruments to measure temporal patterns of neural activity, as they dispose of a very high temporal resolution that reaches milliseconds, despite a much lower spatial resolution (Lin et al., 2018). Finally, physiological peripheral measures comprise a variety of tools, such as *heart rate* (HR; Poels and Dewitte, 2006), *electrodermal activity* (EDA; Ohira and Hirao, 2015), *eye-tracking* (Wedel and Pieters, 2008), and *facial electromyography* (Li et al., 2018; Lajante et al., 2020). These tools have frequently been used in consumer research because they all bear the advantage of being highly cost-effective and easily implemented, providing an online measure of peripheral activation, which lies mostly outside awareness and control.

Researchers approaching the study of consumer behavior through neurophysiological techniques should be well aware of the specificity of each tool, to take into account which technique is most suitable to their research purpose (for review, see Harris et al., 2018; see also Lajante and Ladhari, 2019). Some of the most recent studies seem to have employed neuroscientific tools more consciously, perhaps with a better understanding of the strengths and limits of each technique and methodological flaws (Plassmann et al., 2015). However, fine-grained results are still lacking, and the absence of a “unified theory of decision-making” applied to CN, supported by the joint endeavor of neuroeconomics, neuroscience of decision-making, and consumer research, is hindering the potential of this research field (Sanfey et al., 2006). Thus, the wished methodological turning-point is still far from being completed.

## The Challenges and (Remaining) Concerns of CN

The excitement and renewal of the interest of corporates toward the possibility of unveiling the implicit processes of the mind of the consumer through neuroscientific insights did not run parallel to the dissemination of significant contributions to the field. Some thorough analysis of existing literature (Lee et al., 2018) highlighted the troubling poverty of theoretical discussions and solid methodological primers. The fact that only a few high-quality papers have been published in high-impact journals, whereas an abundance of low-quality overviews is much more easily accessible, undermines the development of the full potential of the discipline and compromises the progress of a scientific theory of CN (Lee et al., 2018).

As a consequence, despite the first attempts to inform the understanding of consumer behaviors with neuroscientific measures trace back to the 1970s (Krugman, 1971; Kroeber-Riel, 1979), CN is still suffering from some of the issues that generally affect unripe research fields, both methodological and theoretical.

The first methodological objection that can be raised toward CN is the extensive use of correlational methods employed for the purpose of mapping the neural basis of specific processes occurring during consumer experience. Neuroimaging techniques, fMRI above all, have been employed in numerous exploratory studies, in an attempt to identify which of the brain regions respond to the exposure to marketing stimuli or perform specific processes that contribute to the final purchase decision. However, neuroimaging techniques do not enable to determine whether the examined cortical structure is causally implied by the ongoing cognitive process or rather its activity is epiphenomenal. Indeed, the only way to ascertain the causal implication of a brain area *Z* in a cognitive process *X* is to demonstrate that (a) activation in area *Z* is necessary for the cognitive process *X* to occur, namely, the cognitive process will not occur unless area *Z* is active or (b) that cognitive process *X* is dose-dependent with respect to the neural activity in area *Z*, namely the higher the activity in brain area *Z*, the more intense (or frequent) the cognitive process *X* (Plassmann et al., 2015).

Therefore, correlational data should be interpreted with caution since this methodological approach engenders several risks. First, it qualifies as a reductionist approach, as it essentially reduces the investigation of consumer decision-making down to a list of associations between brain spots and marketing stimuli, without being informed by theory (Lee et al., 2018; see also Ashkanasy et al., 2014). Secondly, it relies on the outdated assumption that a series of highly functional specialized brain areas exists, which is specifically dedicated to one type of process. Conversely, it has been widely discussed that the brain is much more likely to be organized in the form of complex dynamic networks of interconnected areas, which differentially contribute to the computing and the performing of several cognitive or behavioral processes (Cacioppo et al., 2008).

A second, related challenge, which has long spoiled CN research, is that of *reverse inference* (Hubert and Kenning, 2008; Breiter et al., 2015; Plassmann et al., 2015). The inductive reasoning employed in associative studies has led many researchers to interpret their findings in such a way that the engagement of a psychological process X during an experimental task A is inferred by the observation of neural activation within a specific region Z, which is suggested to be associated to that mental process by other studies (Poldrack, 2006). More plainly:

(1) In the present study, when experimental task A is presented, neural activation is observed in brain region Z.
(2) Previous studies showed that, when cognitive process X is engaged, brain region Z is active.
(3) Hence, the activity of brain region Z in the present study demonstrates that cognitive process X is engaged by the experimental task A.

As we mentioned, reverse inference may be useful in the context of discovery; however, researchers should exercise caution because such logical proceeding cannot discern whether the activation of area Z during task A is selective for the process X. To this end, Poldrack (2006, 2011) suggested a few methodological, statistical, and informatics solutions that can be adopted to properly address reverse inference. Among them, particularly relevant is the possibility to use meta-analytic data of base rates of activation of the investigated area derived from neuroimaging databases to estimate the strength of reverse inferences. To this end, a literature-mining approach was also suggested (Yarkoni et al., 2011). Moreover, to maximize the prior probability that the targeted cognitive process is actually engaged, Poldrack (2006) also endorsed the use of converging evidence from neuroimaging data and behavioral measures; behavioral data could indeed provide additional information, which can further confirm or disconfirm the concurrent activation of the expected psychological process.

## Some Preliminary Conclusions (And Suggestions)

The current state of CN research calls for deeper incorporation of theoretical aspects to inform and interpret results, and caution toward enticing but methodologically problematic inferences. One first step toward more solid results could be represented by a renewed effort to devise a resonate multimethod approach. Systematic integration between behavioral, observational, and neurophysiological data, as well as between different neuroscientific methods, may provide CN with more meaningful findings and contribute to building a broader and more realistic theory of consumer behavior. Neuroimaging data should be interpreted only in the light of supplementing results, collected through behavioral tasks or diverse neurophysiological techniques, and are not to be understood on the mere basis of the putative engagement of a psychological process.

Second, the suggestion that mapping psychological processes onto localized brain regions cannot give account for complex consumer behaviors, has made clear the need for the development of a comprehensive theory of CN. A deep reflection on the theoretical assumptions of the discipline is required; because of its transdisciplinary nature, neuroscientists, neuroeconomists, and scholars from consumer research should contribute to the discussion to address the theoretical foundation of CN. To produce a relevant contribution, CN research needs to be informed by neuroeconomics and decision neuroscience and should invest in testing theories derived by these disciplines to develop an empirically grounded theoretical foundation, rather than exploring little significant brain-tasks associations. In this perspective, future research might clarify whether consumers, when it comes to purchase decisions, do compute the expected value of each alternative to identify the option that maximizes utility (in accordance with the Maximum Expected Utility theory, a central framework of reference in Neuroeconomics) or rather the options are subject to comparative evaluations (Vlaev et al., 2011). Since there is evidence that the brain can switch the modality to neurally represent value according to different purposes and domains (Platt and Padoa-Schioppa, 2009), future research could identify whether different representations are engaged by a distinct type of goods. Relatedly, a relevant issue would be to determine to what extent consumers are capable of estimating costs and benefits of the alternatives in such complex environments before switching to less demanding computing processes, more vulnerable to fallacies (d’Acremont and Bossaerts, 2008).

To sum up, despite progress having certainly been made from its beginnings, CN still needs to carry out a 2-fold effort to finally gain credibility for good. Greater attention should be paid to build a solid methodological framework; on the other hand, research should be employed to validate and possibly expand theory. This way a virtuous cycle of theory-driven experimentation and evidence-based theory development could finally occur and, hopefully, inform corporate practice with meaningful insights.

## Author Contributions

Both authors wrote the first draft and each section of the manuscript, contributed to the manuscript final writing and revision, and read and approved the submitted version.

## Conflict of Interest

The authors declare that the research was conducted in the absence of any commercial or financial relationships that could be construed as a potential conflict of interest.
